# Early Sexual Intercourse: Prospective Associations with Adolescents Physical Activity and Screen Time

**DOI:** 10.1371/journal.pone.0158648

**Published:** 2016-08-11

**Authors:** Raquel Nogueira Avelar e Silva, Anne Wijtzes, Daphne van de Bongardt, Petra van de Looij-Jansen, Rienke Bannink, Hein Raat

**Affiliations:** 1 Department of Public Health, Erasmus University Medical Center, University of Rotterdam, Rotterdam, the Netherlands; 2 Research Institute of Child Development and Education (Research Priority Area YIELD), University of Amsterdam, Amsterdam, the Netherlands; 3 Department of Research and Business Intelligence, Municipality of Rotterdam, Rotterdam, the Netherlands; CHU Clermont-Ferrand, FRANCE

## Abstract

**Objectives:**

To assess the prospective associations of physical activity behaviors and screen time with early sexual intercourse initiation (i.e., before 15 years) in a large sample of adolescents.

**Methods:**

We used two waves of data from the Rotterdam Youth Monitor, a longitudinal study conducted in the Netherlands. The analysis sample consisted of 2,141 adolescents aged 12 to 14 years (mean age at baseline = 12.2 years, *SD* = 0.43). Physical activity (e.g., sports outside school), screen time (e.g., computer use), and early sexual intercourse initiation were assessed by means of self-report questionnaires. Logistic regression models were tested to assess the associations of physical activity behaviors and screen time (separately and simultaneously) with early sexual intercourse initiation, controlling for confounders (i.e., socio-demographics and substance use). Interaction effects with gender were tested to assess whether these associations differed significantly between boys and girls.

**Results:**

The only physical activity behavior that was a significant predictor of early sexual intercourse initiation was sports club membership. Adolescent boys and girls who were members of a sports club) were more likely to have had early sex (OR = 2.17; 95% CI = 1.33, 3.56. Significant gender interaction effects indicated that boys who watched TV ≥2 hours/day (OR = 2.00; 95% CI = 1.08, 3.68) and girls who used the computer ≥2 hours/day (OR = 3.92; 95% CI = 1.76, 8.69) were also significantly more likely to have engaged in early sex.

**Conclusion:**

These findings have implications for professionals in general pediatric healthcare, sexual health educators, policy makers, and parents, who should be aware of these possible prospective links between sports club membership, TV watching (for boys), and computer use (for girls), and early sexual intercourse initiation. However, continued research on determinants of adolescents’ early sexual initiation is needed to further contribute to the strategies for improving adolescents’ healthy sexual development and behaviors.

## Introduction

Early sexual intercourse initiation has been associated with an increased risk of having multiple lifetime sexual partners, unprotected sex, acquiring sexually transmitted infections (STIs), unwanted pregnancy [1–6], and undesirable sexual outcomes, such as problems with orgasm and sexual arousal [4]. In addition, recent studies have found that early sexual intercourse is associated with depression and low self-esteem [7–10]. In light of the risks associated with early sexual intercourse initiation, the understanding of its determinants may contribute to the development and improvement of prevention and intervention strategies and policies aiming to improve adolescents’ sexual health [11]. Established risk factors for early sexual intercourse include low parental educational level, low household income, not living with both biological parents, and poor quality of the parent—adolescent relationship [12–17]. According to the classic ecological model, many other environmental factors (e.g., leisure time activities) may also affect adolescents’ sexual development (e.g., early sexual intercourse initiation) [18]. Although some studies have analyzed the association between adolescents’ physical activity behaviors (e.g., sports participation outside school) and their timing of sexual intercourse [11, 19–24] and one study has analyzed the association between adolescents’ sedentary behavior (e.g., screen time) and their timing of sexual intercourse [25], no studies have examined both behaviors in relation to adolescents’ sexual intercourse initiation.

Overall, regular physical activity has many benefits for adolescents’ physical [26, 27] and psychological health [28], such as improvement of the cardiorespiratory system [26], muscle strength [26], self-esteem [28], and self-confidence [28]. In contrast, sedentary behavior has often been identified as an important risk factor for diseases, such as cardiovascular diseases [29]. However, findings from studies that investigated the associations between physical activity behaviors (e.g., sports participation outside school) and early sexual intercourse are conflicting [11, 19–24, 27, 30]. For instance, one study showed that sports participation outside school was significantly associated with a lower likelihood of having had sexual intercourse [11]. Another study, however, showed that adolescents who participated in sports outside school were significantly more likely to have engaged in sexual intercourse [19]. Other studies showed that girls who participated in sports outside school were less likely to report having had sexual intercourse, whereas for boys no significant association was found [20–23]. One study showed no significant association between sports participation outside school and sexual intercourse [24]. To our knowledge, only one study has investigated the associations between screen time (e.g., computer use) and early sexual intercourse. This cross-sectional study showed that adolescents at higher risk for internet addiction were more likely to have had sexual intercourse [25].

Limitations of previous studies include the cross-sectional designs—only one of them had a prospective design [11]—and therefore evidence on the directionality of the associations is limited. In addition, the majority of these studies were conducted in the United States [11, 19–24]. As cultural aspects, including the sexual double standard and the type of sex education offered in schools (e.g., “comprehensive” versus “abstinence until marriage”) may also influence adolescents’ initiation into sexual intercourse, the research findings from one country may not be generalizable to adolescents living in other countries. For instance, a cross-country comparison of the Netherlands and the United States showed that Dutch adolescents report better sexual health outcomes than American adolescents [31]. A possible explanation could be the longstanding tradition of openness toward adolescent sexuality in the Netherlands [32], which can be seen in the provision of comprehensive sexual education in Dutch high schools [33]. In addition, Dutch parents generally have a more positive and open view of sexuality; initiation into sexual intercourse in adolescence is seen by Dutch parents as a natural path of adolescents’ sexual development [34]. Thus they often provide comprehensive sexual education to their children [35]. Furthermore, none of the aforementioned studies investigated the associations of physical activity behaviors and screen time with early sexual intercourse, i.e., defined by the World Health Organization (WHO) as intercourse initiated before the age of 15 years [1–3]. Yet early sexual intercourse is more likely to constitute a risk behavior than sexual intercourse per se.

The current prospective study aimed to assess the associations of physical activity behaviors and screen time with early sexual intercourse in a large sample of adolescents. Because previous studies investigating the associations between physical activity behaviors and sexual intercourse [11, 19, 24] yielded conflicting results, we had no clear hypothesis regarding these associations. Regarding screen time, we hypothesized that adolescents who reported more daily screen time would be more likely to report early sexual intercourse than adolescents who reported less daily screen time [25].

Furthermore, we were interested in ascertaining gender differences, as previous studies have demonstrated that adolescents’ sexual development, including the initiation of sexual intercourse, differs between boys and girls [36–41]. In the Netherlands, for instance, a study showed that boys initiate sexual intercourse significantly earlier than girls [41]. These gender differences in adolescents’ sexual intercourse initiation might be due to various reasons, including cultural aspects regarding sexuality (e.g., the sexual double standard) [36–39]. In many Western cultures, boys generally have more sexual freedom and are under more pressure from a young age onwards to form and prove their masculinity through sexual activity, whereas girls experience more sexual restrictions [36–39]. This sexual double standard could help explain why in many Western societies boys initiate sexual intercourse earlier than girls [36–39]. To take account of these gender differences in adolescent sexual development, in the current study we assessed the prospective associations of physical activity behaviors and screen time with early sexual intercourse for boys and girls separately, and also tested whether these associations differed between boys and girls.

## Methods

### Study Design

A prospective study was conducted using data from the Rotterdam Youth Monitor (RYM). This is a longitudinal youth health surveillance system that is incorporated into the preventive youth healthcare system of Rotterdam [42]. Its aim is to monitor the health, well-being, and behaviors of children and adolescents, to detect potential health risks and problems, and to apply preventive measures [42]. During the years of 2008–2009 (T1) and 2010–2011 (T2) the RYM conducted a prospective study in secondary schools in Rotterdam and surrounding regions. Data were collected from a community sample of 8,272 adolescents at baseline and 3,184 adolescents at follow-up. For the current study, data from both waves were used, with a two-year interval between the measurements. At T1, 76 secondary schools (100% school participation rate) and 8,272 students enrolled in the first year of secondary school (95% student participation rate) participated in the study. The main reason for non-response at baseline was students’ illness when the questionnaires were being administered. At follow-up, 45 of the schools (59% school participation rate) and 3,184 students who by now were enrolled in the third year of secondary school (38% student participation rate) participated in the study again. The main reason for non-response at follow-up was that some schools were unwilling to participate in the study again. Other reasons included students’ absence when the follow-up questionnaire was being administered, students’ transfer to another school that did not participate at follow-up, and students repeating a school year. Data were collected throughout the school year, except during the summer vacation months of July and August. Administration of the questionnaires at schools was guided by trained researchers, school nurses from the Municipal Public Health Service, and teachers.

### Study Sample

For our analysis, we selected only students who participated at both T1 and T2 (n = 3,184). In addition, to be able to predict the initiation of early sexual intercourse, i.e., intercourse experience before the age of 15 years [1–3, 43], we selected only participants who were younger than 15 years old at both T1 and T2 (n = 1,001 excluded) and who at T1 had never had sexual intercourse (n = 26 excluded). Furthermore, we excluded participants with missing information on sexual intercourse at T1 (n = 7) and/or T2 (n = 9). This resulted in 2,141 adolescents included in the prospective analysis sample.

### Ethics Statement

All data were collected within the government-approved routine health examinations of the Rotterdam preventive youth healthcare system. Observational research with anonymous data gathered in routine health examinations of this preventive youth healthcare system does not fall within the ambit of the Dutch Act on research involving human subjects, and does not require the approval of an ethics review board. The data of the Rotterdam Youth Monitor Study is protected by the Municipal Health Service of Rotterdam, which follows the Code of Conduct Health Research of the Netherlands. All records/information were anonymized and de-identified prior to analysis. The questionnaires were completed voluntarily, and confidentiality of responses was guaranteed. Adolescents received verbal information about these questionnaires each time they were administered; their parents received written information at every assessment point. Adolescents and their parents were free to refuse to participate. The data became available in the context of the government-approved routine health examinations of Rotterdam’s preventive youth healthcare. Separate informed consent was therefore not required [42].

### Measures

A detailed description of the original variables can be found in S1 Table.

#### Physical activity behaviors

Physical activity behaviors were assessed by a set of four questions in the self-report questionnaire on: 1) cycling to school; 2) time cycling to school; 3) sports club membership; and 4) sports participation outside school. Cycling to school was measured using one item: “How many days per week do you cycle to school?”, (0 = Never; 1 = 1 day; 2 = 2 days; 3 = 3 days; 4 = 4 days; 5 = 5 days). This variable was dichotomized (0 = Never or 1 = Ever). The time cycling to school was measured using one item “How long does it take you to cycle to and from school?”, (1 = Zero; 2 = <10 minutes; 3 = 10–20 minutes; 4 = 20–30 minutes; 5 = 30 minutes-1 hour; 6 = >1 hour). This variable was dichotomized (0 = <30 minutes/day; 1 = ≥30 minutes/day). Sports participation outside school was also measured using one item “How many days per week do you participate in sports outside school?”, (0 = 0 days; 1 = 1 day; 2 = 2 days; 3 = 3 days; 4 = 4 days; 5 = 5 days; 6 = 6 days; 7 = 7 days). This variable was categorized into three categories (0 = Never; 1 = 1–3 days/week; 2 = 4–7 days/week).

#### Screen time

Screen time questions related to Television/Digital Versatile Disc (TV/DVD) watching and computer use (games, internet). TV/DVD watching was measured using one item “How many hours per day do you watch TV/DVD?”, (0 = zero hours; 1 = 1 hour; 2 = 2 hours; 3 = 3 hours; 4 = 4 hours; 5 = 5 hours). Computer use was also measured using one item “How many hours per day do you use the computer (e.g., for games or internet)?”, (0 = zero hours; 1 = 1 hour; 2 = 2 hours; 3 = 3 hours; 4 = 4 hours; 5 = 5 hours). These variables were dichotomized (0 = <2 hours/day or 1 = ≥2 hours/day) [44, 45].

#### Early sexual behavior

Early sexual intercourse was defined as intercourse initiated before the age of 15 years as proposed by the WHO [1–3]. This variable was measured using one item “Have you ever had sexual intercourse? (by sexual intercourse we mean penile—vaginal intercourse)”, (0 = No, never; 1 = Yes, one time; 2 = Yes, a couple of times; 3 = Yes, regularly). This variable was dichotomized (0 = Never; 1 = Ever).

#### Potential confounders

Based on previous studies, the following variables were considered potential confounders in the associations of physical activity behaviors and screen time with early sexual intercourse: gender, age, educational level, ethnic background, family structure, smoking, alcohol use, and marijuana use. Secondary school students enrolled in basic or theoretical pre-vocational education (VMBO) were classified as low educational level, whereas students enrolled in senior general education (HAVO) or pre-university education (VWO) were classified as high educational level [42, 46]. In accordance with the definition used by Statistics Netherlands, adolescents were considered non-native Dutch if at least one of their parents had been born abroad [42, 46].

### Statistical Analyses

Descriptive statistics were used to portray the characteristics of the study sample. Prospective associations of physical activity behaviors and screen time with early sexual intercourse were assessed by a series of logistic regression models, stratified by gender.

First, a model was tested containing the confounders (i.e., gender, age, educational level, ethnic background, family structure, smoking, alcohol and marijuana use) and the physical activity behaviors (i.e., Model 1). Second, a model was tested containing the confounders and the screen time variables (i.e., Model 2). A third model was tested containing the confounders, the physical activity behaviors, and the screen time variables simultaneously (i.e., Model 3). In addition, we assessed *model chi-square statistics* for all models (i.e., Models 1, 2, and 3) and tested the difference in these statistics between Models 1 and 3, and between Models 2 and 3, to assess which model had the best fit in the stratified analysis. *Model chi-square statistics* show how much the model is improved after new variables have been added to the model. When the difference between two model fits is significant (*p*<0.05), the model with the highest *chi-square* values best fits.

After the stratified regression analyses by gender, we also tested gender interaction effects (i.e., gender x physical activity, gender x screen time, and gender x confounders) using the model with the best fit, and using data from the total study sample (not stratified). All analyses were conducted in 2015 with the Statistical Package for Social Sciences (SPSS) version 21.0 for Windows (IBM Corp., Armonk, NY, USA) and using a significance level of *p*<0.05.

### Non-response Analysis

Adolescents who were included in the prospective analysis sample (n = 2,141) were compared with those who were excluded (n = 6,131), using chi-square tests. The results of these tests showed that, compared with the adolescents included in the analysis sample, the excluded adolescents were more often boys (*X*^2^ = 14.2, *df* = 1, *p* < .001), more often had a low educational level (*X*^2^ = 420.0, *df* = 1, *p* < .001), were more often native Dutch (*X*^2^ = 7.3, *df* = 2, *p* < .05), more often lived with both parents (*X*^2^ = 57.0, *df* = 1, *p* < .001), more often cycled to school (*X*^2^ = 10.5, *df* = 1, *p* < .001), more often cycled to school <30 minutes/day (*X*^2^ = 44.1, *df* = 1, *p* < .001), more often watched TV ≥2 hours/day (*X*^2^ = 80.1, *df* = 1, *p* < .001), and more often used a computer ≥2 hours/day (*X*^2^ = 85.1, *df* = 1, *p* < .001). Differences between the two groups in sports club membership *(X*^*2*^ = 1.1, *df* = 1, *p* = .150) and sports participation outside school *(X*^*2*^ = 3.0, *df* = 2, *p* = .220) were not statistically significant.

## Results

### Characteristics of the Study Sample

At baseline, the mean age of the adolescents was 12.2 years (*SD* = 0.43). Additional characteristics of the study sample at baseline and the differences between boys and girls can be seen in Table 1. Several significant gender differences were found. Girls were more likely than boys to be non-native Dutch (*p* < .001), to report not living with both biological parents (*p* < .001), not to be members of a sports club (*p* < .001), and to never participate in sports outside school (*p* < .001). Regarding adolescents’ early initiation into sexual intercourse, boys were more likely than girls to report having had experience with sexual intercourse at T2 (*p* < .001).

**Table 1:** Descriptive characteristics of the prospective analysis sample, and gender differences, at baseline (n = 2,141).

	Total		Boys		Girls		*p* ^a^
	n	%	n	%	n	%	
*Confounders*							
Gender	2,141	100.0	1,030	48.1	1,111	51.9	-
Age							
11–12 years	1,730	80.8	833	48.2	897	51.8	**< .001**
Educational Level							
Low	852	39.8	393	46.1	459	53.9	.140
Ethnic background							
Non-native Dutch	956	44.7	428	44.8	528	55.2	**< .001**
Family Structure							
Not living with both biological parents	440	20.6	182	41.4	258	58.6	**< .001**
Smoking							
Yes	196	9.2	106	54.1	90	45.9	.081
Alcohol use							
Yes	201	9.4	106	52.7	95	47.3	.180
Marijuana use							
Yes	6	0.3	4	66.7	2	33.3	.364
*Physical activity behaviors*							
Cycling to school							
Never	416	19.4	192	46.2	224	53.8	.380
Time cycling to school							
<30 minutes/day	1,486	69.6	716	48.2	770	51.8	.940
Sports club membership							
No	769	36.0	286	37.2	483	62.8	**< .001**
Sports outside school							
Never	226	10.6	63	27.9	163	72.1	**< .001**
*Screen time*							
TV/DVD watching							
≥2 hours/day	1,209	56.5	467	50.3	462	49.7	.083
Computer use							
≥2 hours/day	1,047	49.0	519	47.6	572	52.4	.630
*Sexual Behavior*							
Early sexual intercourse at T2							
Ever	129	6.0	79	61.2	50	38.8	**< .001**

^a^ Significance levels of differences in characteristics measured at T1 between boys and girls by Chi-Square tests (categorical variables).

Note: Non-native Dutch included: Surinamese, Turkish, Dutch Antillean, Moroccan, Cape Verdean.

### Prospective Associations of Physical Activity Behaviors and Screen Time with Early Sexual Intercourse Initiation

Table 2 shows the logistic regression analyses of the associations of physical activity and screen time at baseline with early sexual intercourse at follow-up, stratified by gender. Results of *chi-square difference tests* (Table 2) showed that Model 3 had a significantly better fit than Models 1 and 2, both for boys (Δχ^2^_1–3_ (df) = 2; *p* = .002), (Δχ^2^_2–3_ (df) = 5; *p* = .032), and for girls (Δχ^2^_1–3_ (df) = 2; *p* = .006). Therefore, the gender interaction effects were tested in Model 3, and the results hereof are presented in Table 3.

**Table 2:** Results of logistic regression analyses of the prospective associations of physical activity behaviors and screen time with early sexual intercourse, stratified by gender (n = 2,141).

	Early sexual intercourse					
	Model 1^a^		Model 2^b^		Model 3^c^	
	OR (95% CI)		OR (95% CI)		OR (95% CI)	
	Boys (n = 1,023)	Girls (n = 1,094)	Boys (n = 1,025)	Girls (n = 1,097)	Boys (n = 1,020)	Girls (n = 1,091)
*Confounders*						
Age (0 = 11–12 years)						
13–14 years	1.45 (0.83, 2.54)	0.84 (0.37, 1.92)	1.36 (0.78, 2.36)	0.80 (0.35, 1.81)	1.40 (0.80, 2.46)	0.84 (0.36, 1.93)
Educational level (0 = High)						
Low	2.51 (1.52, 4.15)***	0.80 (0.40, 1.60)	2.17 (1.33, 3.57)**	0.75 (0.38, 1.46)	2.29 (1.38, 3.81)**	0.77 (0.38, 1.55)
Ethnic background (0 = Native Dutch)						
Non-native Dutch	1.68 (0.98, 2.88)	0.77 (0.39, 1.55)	1.49 (0.90, 2.46)	0.74 (0.39, 1.39)	1.47 (0.85, 2.54)	0.77 (0.38, 1.57)
Family Structure (0 = Living with both biological parents)						
Not living with both biological parents	1.95 (1.12, 3.39)*	2.31 (1.21, 4.40)*	1.74 (1.00, 3.01)	2.18 (1.15, 4.12)*	1.96 (1.12, 3.43)*	2.14 (1.12, 4.09)*
Smoking (0 = No)						
Yes	1.73 (0.89, 3.37)	7.56 (3.61, 15.79)***	1.70 (0.88, 3.28)	6.24 (3.02, 12.86)***	1.62 (0.84, 3.16)	6.12 (2.91, 12.83)***
Alcohol use (0 = No)						
Yes	2.30 (1.18, 4.47)*	1.62 (0.70, 3.74)	2.05 (1.06, 3.97*)	1.29 (0.57, 2.94)	2.14 (1.09, 4.19*)	1.43 (0.61, 3.35)
Marijuana use (0 = No)						
Yes	3.80 (0.45, 32.28)	**-**	7.36 (0.85, 63,82)	**-**	5.44 (0.60, 49.19)	**-**
*Physical activity behaviors*						
Cycling to school (0 = Never)						
Ever	0.62 (0.35, 1.11)	0.96 (0.43, 2.20)			0.65 (0.36, 1.16)	1.00 (0.45, 2.26)
Time cycling to school (0 = <30 min/day)						
≥ 30 minutes/day	0.92 (0.50, 1.69)	0.60 (0.28, 1.27)			1.03 (0.56, 1.92)	0.64 (0.30, 1.39)
Sports club membership (0 = No)						
Yes	1.90 (1.00, 3.59)	1.73 (0.79, 3.79)			1.96 (1.03, 3.72)	1.69 (0.77, 3.71)
Sports outside school (0 = Never)						
1–3 days	0.80 (0.24, 2.68)	0.48 (0.18, 1.30)			0.89 (0.26, 3.00)	0.53 (0.20, 1.43)
4–7 days	1.03 (0.32, 3.37)	0.67 (0.24, 1.82)			1.16 (0.36, 3.82)	0.74 (0.27, 2.02)
*Screen Time*						
TV/DVD watching (0 = <2 hours/day)						
≥2 hours/day			2.14 (1.18, 3.90)*	0.68 (0.35, 1.32)	2.00 (1.08, 3.68)*	0.70 (0.35, 1.39)
Computer use (0 = <2 hours/day)						
≥2 hours/day			1.24 (0.75, 2.06)	4.30 (1.95, 9.44)***	1.35 (0.80, 2.28)	3.92 (1.76, 8.69)**
Model ^d^	*X*^2^ (12) = 55.34***	*X*^2^ (12) = 53.96***	*X*^2^ (9) = 54.92***	*X*^2^ (9) = 63.48***	*X*^2^ (14) = 67.10***	*X*^2^ (14) = 64.00***

Note: Bold print indicates statistical significance. Reference groups are equal to zero.

*p < .05,

**p < .01,

***p < .001.

^a^ Model 1 included confounders (i.e., gender, age, educational level, ethnic background, family structure, smoking, alcohol, and marijuana use) and physical activity behaviors.

^b^ Model 2 included confounders and screen time variables.

^c^ Model 3 included confounders and physical activity behaviors and screen time variables simultaneously.

^d^ Assessment of the model fit, using chi-square difference tests.

**Table 3:** Results of logistic regression analyses of the prospective associations of physical activity behaviors and screen time with early sexual intercourse, for the total study sample (n = 2,141).

Early sexual intercourse	
	Model 3 ^a^
	OR (95% CI)
	(n = 2,111)
*Confounders*	
Gender (0 = Girls)	
Boys	1.73 (1.16, 2.59)**
Age (0 = 11–12 years)	
13–14 years	1.19 (0.75, 1.88)
Educational level (0 = High)	
Low	1.60 (1.07, 2.38)*
Ethnic background (0 = Native Dutch)	
Non-native Dutch	1.20 (0.79, 1.83)
Family Structure (0 = Living with both biological parents)	
Not living with both biological parents	2.04 (1.35, 3.08)**
Smoking (0 = No)	
Yes	2.75 (1.70, 4.42)***
Alcohol use (0 = No)	
Yes	1.82 (1.09, 3.05)*
Marijuana use (0 = No)	
Yes	2.03 (0.29, 14.20)
*Physical activity behaviors*	
Cycling to school (0 = Never)	
Ever	0.78 (0.49, 1.23)
Time cycling to school (0 = <30 min/day)	
≥ 30 minutes/day	0.87 (0.54, 1.40)
Sports club membership (0 = No)	
Yes	2.17 (1.33, 3.56)**
Sports outside school (0 = Never)	
1–3 days	0.58 (0.28, 1.21)
4–7 days	0.80 (0.39, 1.66)
*Screen time*	
TV/DVD watching (0 = <2 hours/day)	
≥2 hours/day	1.28 (0.82, 1.99)
Computer use (0 = <2 hours/day)	
≥2 hours/day	1.98 (1.29, 3.02)**

Note: Bold print indicates statistical significance.

*p <0.05,

**p <0.01,

***p <0.001

^a^ Model 3 included confounders (i.e., gender, age, educational level, ethnic background, family structure, smoking, alcohol, and marijuana use), and physical activity behaviors and screen time variables simultaneously.

With regard to the potential confounders, Model 3 (Table 2) revealed that boys (but not girls) with low educational level were significantly more likely to have engaged in early sexual intercourse between T1 and T2 than boys with high educational level (OR = 2.29; 95% CI = 1.38, 3.81). In addition, Model 3 showed that boys and girls who were not living with both biological parents were significantly more likely to have engaged in early sexual intercourse between T1 and T2 than boys and girls who were living with both biological parents (OR_boys_ = 2.29; 95% CI = 1.38, 3.81), (OR_girls_ = 2.29; 95% CI = 1.38, 3.81). Model 3 also showed that girls (but not boys) who smoked were significantly more likely to have engaged in early sexual intercourse between T1 and T2 than girls who did not smoke (OR = 6.12; 95% CI = 2.91, 12.83). Furthermore, Model 3 showed that boys (but not girls) who drank alcohol were significantly more likely to have engaged in early sexual intercourse between T1 and T2 than boys who did not drink alcohol (OR = 2.14; 95% CI = 1.09, 4.19). The analyses for the total sample revealed significant interaction effects between gender and educational level (*p* = .010), and between gender and smoking (*p* = .009), indicating that these statistics were significantly different for boys and girls.

As can be further seen in Table 2, Model 3 revealed that for boys and for girls, none of the physical activity behaviors were significantly associated with early sexual intercourse initiation. We also found no significant interaction effects between gender and physical activity behaviors. Our results did show that sports club membership was a significant predictor of sexual intercourse initiation. Adolescents (both boys and girls) who were members of a sports club were significantly more likely to have had early sexual intercourse (OR = 2.17; 95% CI = 1.33, 3.56) than adolescents who were not members of a sports club (Table 3).

Model 3 also revealed that the screen time variables significantly associated with early sexual intercourse initiation were TV watching (for boys) and computer use (for girls). We found two significant interaction effects between gender and TV watching (for boys only), (*p* = .026), and between gender and computer use (for girls only), (*p* = .030). Boys who watched TV ≥2 hours/day were significantly more likely to have engaged in early sexual intercourse between T1 and T2 than boys who watched TV <2 hours/day (OR = 2.00; 95% CI = 1.08, 3.68). Girls who used a computer ≥2 hours/day were significantly more likely to have engaged in early sexual intercourse between T1 and T2 than girls who used a computer <2 hours/day (OR = 3.92; 95% CI = 1.76, 8.69).

## Discussion

The present study aimed to assess the prospective associations of physical activity behaviors and screen time with early sexual intercourse (i.e., sexual intercourse before 15 years) in a large population of adolescents.

### Gender Differences

In our study, a relatively small percentage of adolescents (i.e., 6.0%) initiated sexual intercourse between T1 and T2. This may be explained by the relatively young age of our participants (i.e., M_*age*_ = 12.2 years at T1). In the Netherlands, the average age at which adolescents initiate sexual intercourse is 16.6 years [41]. The fact that boys in our young sample were more likely to engage in early sexual intercourse than girls is consistent with previous studies from different countries, including the Netherlands [41] and the United States [19, 23, 47]. This gender difference may be related to our use of self-reports about sexual behavior. The reliability of this method may be questioned, as boys are known to often over-report and girls to often under-report their sexual activities [48]. Alternatively, it may be related to the still existing sexual double standard. Recent studies on the sexual double standard during adolescence have shown that in many societies, girls have gained more sexual freedom in recent decades. But despite this progress, the sexual double standard still exists, and in a range of settings (e.g., schools), girls who start sexual intercourse at an early age may still be subjected to negative social sanctions or restrictions [36–39]. In another direction, these studies have shown that boys do indeed have more sexual freedom than girls, and tend to gain a better reputation after they start having sexual intercourse. For boys, early sexual intercourse may thus be related to a need to prove their masculinity [23], whereas for girls, early sexual intercourse may come at a higher social cost (e.g., a bad reputation), and for this reason they may avoid engaging in such behavior at an early age [49]. This well-recognized historical and cultural phenomenon of the sexual double standard may thus help explain our finding that boys were more likely than girls to have engaged in early sexual intercourse.

### Physical Activity Behaviors and Early Sexual Intercourse Initiation

We found that, in the total study sample, the only significant predictor of early sexual intercourse was sports club membership, whereas the other physical activity behaviors (e.g., sports participation outside school) were not. Similarly, a cross-sectional study conducted in the United States also found no association between sports participation outside school and early sexual intercourse [24]. Findings from studies that investigated the associations between physical activity behaviors and sexual intercourse are conflicting; most of these studies were cross-sectional in design [19–24] and were conducted in the United States [11, 19–24]. Thus, to elucidate the role of physical activities on adolescents’ early sexual intercourse initiation, it would be worth investigating the longitudinal associations between specific types of sports and early sexual initiation, and to do so in different countries.

In our total study sample, adolescents who were members of a sports club were significantly more likely to have engaged in early sexual intercourse than adolescents who were not members of a sports club. A possible explanation for our finding could be that adolescents who play sports in the setting of a sports club may be more likely to spend considerable unsupervised leisure time with peers than those who practice sports in a different setting. A sports club may not only be a place where adolescents practice physical activities, but it may also be a place where adolescents interact with potential sexual partners (e.g., in the bar of the sports club), which could facilitate early sexual experiences. Prospective studies conducted in United States have indeed demonstrated that adolescents who spent more unsupervised time with their peers were more likely to engage in sexual intercourse [19, 50, 51].

Although was expected that the associations between physical activity behaviors and early sexual intercourse might differ for boys and girls [20–23], in the present study we did not find significant interaction effects between adolescents’ gender and physical activity behaviors on early sexual intercourse. Given that only a small number of boys and girls initiated sexual intercourse between T1 and T2, the lack of significant interaction effects might be the result of the lack of statistical power. Future studies should assess the associations between physical activity behaviors and adolescents’ early sexual intercourse initiation in larger and older samples.

### Screen Time and Early Sexual Intercourse Initiation

The finding that boys (but not girls) who watched TV ≥2 hours/day were significantly more likely to have had early sexual intercourse than boys who watched TV <2 hours/day is similar to findings of previous studies that showed that a greater exposure to sexual content on TV/DVDs predicted sexual intercourse among adolescents (girls and boys) [47, 52, 53]. However, to our knowledge, ours is the first study showing these associations with early sexual intercourse (i.e., before the age of 15 years). Prevalence of sexual content in TV programs may vary across countries, but watching TV ≥2 hours/day may increase the level of exposure to sexual content. Thus a possible explanation for our finding could be that an increased exposure to sexual content may cause adolescents to believe that sexual intercourse is a normative part of everyday life, which could be a stimulus for sexual intercourse [47]. The fact that TV watching ≥2 hours/day was associated with an increased risk of early sexual intercourse for boys only, and not for girls, may be explained by the co-viewing status. For instance, a study found that adolescents who watched TV at least once per week with a peer of the opposite sex were significantly more likely to engage in sexual intercourse than adolescents who did not watch TV weekly with a friend of the opposite sex [52]. The presence of an opposite-sex peer could strengthen the influence of media (sexual content), and perhaps boys are more susceptible to media exposure [52]. However, this is difficult to ascertain because in our analysis we did not control for TV co-viewing status. An additional explanation for our finding may be a lack of parents’ control over the media consumption of their adolescent children [11, 54]. This notion is supported by a prospective study showing that adolescents whose parents restricted their TV watching were less likely to report early sexual intercourse [54].

We also found that girls who used a computer ≥2 hours/day were significantly more likely to have engaged in early sexual intercourse than girls who used a computer <2 hours/day, whereas for boys computer use did not affect early sexual initiation. This is partially in line with the finding of a cross-sectional study that showed that adolescents at high risk for internet addiction (i.e., an indicator of screen time/computer use) were significantly more likely to have had sexual intercourse [25]. A possible explanation for our finding could be that girls who are greatly exposed to computer use (e.g., high-frequent internet use) may be more likely to contact potential sexual partners online, as well as to be solicited online for sex more than boys who are highly exposed to computer use [55]. We do not know, however, what the exact reasons were for girls’ computer use (e.g., homework, gaming, or viewing sexualized internet material). This is a relevant area for future studies.

### Strengths and Limitations

To our knowledge, ours is the first prospective study to assess the associations of physical activity behaviors and screen time (separately and simultaneously) with early sexual intercourse in a large sample of adolescents. Besides assessing gender differences, we were able to control for a wide range of potential confounders that have been associated with sexual intercourse. However, some limitations should be taken into account when interpreting the results. First, information on all variables was assessed by self-report questionnaires, which may have led to socially desirable answers. Second, early sexual intercourse was measured as a single indicator of sexual risk behavior. Third, our findings are difficult to compare with findings from other studies conducted in other countries because not all studies have used the WHO definition of early sexual intercourse that we used. Finally, our non-response analyses comparing participants and non-participants in the present study, showed differences in sociodemographic characteristics, physical activity behaviors, and screen time. Although selective participation is a common problem in longitudinal research on adolescents sexual development [56], it may have affected our results. However, it is difficult to ascertain the consequences of selective participation because some features of non-participants could be considered a risk factor (e.g., excluded adolescents more often had low educational level at T1), whereas other features could be considered a protective factor (e.g., excluded adolescents were more often living with both biological parents at T1).

## Conclusions

The present study shows that for the Dutch adolescents in our sample (both boys and girls), the only significant predictor of early sexual intercourse (i.e., before the age of 15 years, as proposed by the WHO) was sports club membership. Adolescents who were members of a sports club were significantly more likely to have had early sex. However, some predictors of early sexual intercourse were gender-specific. Specifically, boys who watched TV ≥2 hours/day and girls who used a computer ≥2 hours/day were significantly more likely to have engaged in early sexual intercourse. These findings have implications for professionals in general pediatric healthcare, sexual health educators, policy makers, and parents, who should be aware of these possible prospective links between early sexual intercourse and sports club membership, and for boys, TV watching, and for girls, computer use. However, more research is needed to better understand the mechanisms underlying these associations, for instance by more closely exploring the socio-contextual factors in sports clubs that may contribute to opportunities for early sexual activities. Furthermore, the assessment of the associations between different features of sports (e.g., individual versus collective) and early sexual initiation may also provide more evidence and understanding about whether the type of sports adolescents play is relevant in relation to their early initiation into sexual intercourse. It is also important to conduct further longitudinal studies examining the over-time associations of physical activity behaviors and screen time with sexual development, and to improve our understanding of possible gender and age differences therein. Another important direction for future investigations is the assessment of the associations of physical activities behaviors and screen time with early sexual intercourse in different countries, because socio-cultural aspects (e.g., the provision of “comprehensive” versus “abstinence until marriage” sex education) may also influence the effects of physical activities and screen time on the initiation of early sexual intercourse. Continued research on determinants of adolescents’ early sexual intercourse initiation is needed to further contribute to the strategies aimed at the improvement of adolescents’ healthy sexual development and behaviors.

## Supporting Information

S1 Table Description of the original variables. (PDF)
