# Supplementary material for: Early Sexual Intercourse: Prospective Associations with Adolescents Physical Activity and Screen Time
Source: PLoS One. 2016 Aug 11;11(8):e0158648. doi: 10.1371/journal.pone.0158648 (PMC4981454; doi:10.1371/journal.pone.0158648)
Supplement: S1 Table — (PDF) [file pone.0158648.s001.pdf]

S1 Table. Description of the original variables.

Variables	Items	Questions	Response options	Cutt-offs dichotomous
Physical Activity Behaviors	Cycling to school	How many days/week do go to school by bike?	Zero	Never
			1 day	Ever
			2 days	
			3 days	
			4 days	
			5 days	
	Time cycling to school	How long does it take you to cycle to and from school?	< 10 minutes	
			10-20 minutes	
			20-30 minutes	< 30 minutes/day
			30 minutes-1 hour	≥ 30 minutes/day
			> 1 hour	
	Sports club membership	Are you a member of a sports club?(i.e., whether adolescents had a paid membership of a sports club).	No	No
			Yes	Yes
	Sports outside school	How many days/week do you participate in sports outside school?	Zero	Never
			1 day	
			2 days	
			3 days	1-3 days/week
			4 days	4-7 days/week
			5 days	
			6 daya	
			7 days	
Screen Time	TV/DVD watching	How many hours/day do you watch TV/DVDs?	Zero	
			< 1 hour	
			1 hour	< 2 hours/day
			2 hours	≥ 2 hours/day
			3 hours	
			4 hours	
			5 hours	

Variables	Items	Questions	Response options	Cutt-offs dichotomous
Screen Time	Computer use	How many hours/day do you use a/the? computer (games, internet)?	Zero	
			< 1 hour	
			1 hour	< 2 hours/day
			2 hours	≥ 2 hours/day
			3 hours	
			4 hours	
			5 hours	
Sexual Behavior	Early sexual intercourse	Have you ever had sexual intercourse (by sexual intercourse we mean penile-vaginal intercourse)?	No, never	Never
			Yes, 1 time	Ever
			Yes, a couple of times	
			Yes, regularly	
Potential Confounders	Gender	Are you a boy or a girl?	Boy	Boy
			Girl	Girl
	Age	How old are you?	11 years	
			12 years	11-12 years
			13 years	13-14 years
			14 years	
			15 years	Excluded
			16 years	
			17 years	
			≥ 18 years	
	Educational Level	Indicate in which class you are and what kind of educational level you follow	VMBO-vocational	
			VMBO-theoretical	
			VMBO/HAVO	Low
			HAVO/VWO	High
			VWO	
	Ethnic Background	In what country were you born?	the Netherlands	Native-Dutch
			Suriname	Non-native Dutch
			Dutch Antilles or Aruda	
			Morocco	
			Turkey	
			Cape Verde	
			Other	

Variables	Items	Questions	Response options	Cutt-offs dichotomous
Potential Confounders	Family Structure	Do you live with your mother and father?	Yes, I live with my father and mother<br>Yes, but sometimes I live with my mother and sometimes with my father<br>No, I live with my father<br>No, I live with my mother<br>No, I live with my father and stepmother/friend<br>No, I live with my mother and stepfather.friend<br>No, I live with other	Living with both biological parents<br>Not living with both biological parents
	Smoking	Have you ever smoked (cigarettes)?	No, I have never smoked<br>Yes, I smoked 1 or 2 times<br>I smoke occasionally<br>I smoke daily	No<br>Yes
	Alcohol	Do you sometimes drink alcohol?	No, never<br>Yes, sometimes	No<br>Yes
	Marijuana	Have you ever used marijuana?	Never<br>1-2 times<br>3-4 times<br>5-6 times<br>7-10 times	No<br>Yes
